# The 72-Item Semi-Quantitative Food Frequency Questionnaire (72-Item SQ-FFQ) for Polish Young Adults: Reproducibility and Relative Validity

**DOI:** 10.3390/nu14132696

**Published:** 2022-06-29

**Authors:** Joanna Kowalkowska, Lidia Wadolowska

**Affiliations:** Department of Human Nutrition, The Faculty of Food Science, University of Warmia and Mazury in Olsztyn, Słoneczna 45F, 10-718 Olsztyn, Poland; lidia.wadolowska@uwm.edu.pl

**Keywords:** food frequency questionnaire, dietary record, food record, reliability, validity, food consumption, nutrient intake, macronutrients, micronutrients, adults

## Abstract

The food frequency questionnaires (FFQs) are the most common tools used in dietary research. Each newly developed, modified, or adapted FFQ should be validated in the target population. The aim of this study was to assess the reproducibility and relative validity of the 72-item semi-quantitative food frequency questionnaire (72-item SQ-FFQ) for Polish adults. The 72-item SQ-FFQ was developed based on a non-quantitative FFQ covering 62 food items (62-item FFQ-6®). The study was conducted among 186 university students aged 19–26 years (47.8% of females). The FFQ was administered on two occasions (FFQcrude and FFQretest) to assess the test–retest reproducibility, and the FFQcrude was compared with the estimated food record (FRcrude) to evaluate the relative validity of the FFQ in assessing the intake of energy, 38 nutrients, and alcohol. The energy intake obtained with both methods was standardized to 2000 kcal/day (FFQstand, FRstand). The dietary intake obtained with FFQcrude was adjusted using linear regression analysis (FFQreg). The reproducibility and relative validity of the FFQ were assessed by comparing the mean values of energy and nutrient intake and using Spearman’s correlation coefficient, the cross-classification analysis, and the Bland–Altman method. Spearman’s correlation coefficient between both administrations of the FFQ ranged from 0.631 to 0.878 (the intraclass correlation coefficient: 0.583–0.935), for FFQcrude and FRcrude ranged from −0.025 to 0.390, for FFQstand and FRstand ranged from 0.021 to 0.546, and for FFQreg and FRcrude ranged from 0.028 to 0.391. The percentage of respondents classified into the same or adjacent quartiles of nutrient intake obtained from two administrations of the FFQ ranged from 84.9% to 97.8%, for FFQcrude and FRcrude ranged from 61.3% to 76.9%, for FFQstand and FRstand ranged from 63.4% to 83.9%, and for FFQreg and FRcrude ranged from 60.2% to 76.9%. In conclusion, our findings showed good reproducibility and acceptable relative validity of the 72-item SQ-FFQ. This tool can be recommended for assessing dietary intake among Polish young adults. However, the intake of certain nutrients should be interpreted with caution.

## 1. Introduction

The food frequency consumption is the most commonly used dietary assessment method in epidemiological studies [1,2,3]. Food frequency questionnaires (FFQs) are usually self-administered. Besides the food list and the consumption frequency category section (non-quantitative FFQs), the FFQs may include the portion size of each food item (semi-quantitative FFQs) [3]. The FFQs are relatively inexpensive and related to low respondent burden; therefore, they are appropriate tools for large studies. However, the food list included in an FFQ cannot cover all the foods that may be consumed by a respondent, especially novel foods [2,3]. A long food list in the FFQ increases respondent burden [2,4]. Moreover, inappropriate food grouping can lead to an overestimation of dietary intake [4,5]. Filling in the semi-quantitative FFQs, respondents can have difficulties with the estimation of the portion size of consumed food. Portion sizes of foods of irregular shape (e.g., lettuce, pasta) can be more difficult to estimate than foods purchased and/or consumed regularly in defined amounts (e.g., bread in slices, beverages in bottles) [2].

Each newly developed, modified, or adapted food frequency questionnaire (FFQ) should be validated in the target population [1,4,5]. The commonly used reference methods to calibrate the FFQ are weighted food record, estimated food record, and 24 h dietary recall [5]. The retrospective methods, such as the food frequency method and the 24 h dietary recall, depend on the respondent’s memory and ability to recall the consumed foods, consumption frequency, and/or portion sizes over a certain period of time [2,3]. When assessing the validity, the tested method should not have similar disadvantages to the reference method [5]. The measurement errors of both methods used should be independent. The prospective methods, such as both estimated and weighted food records, do not rely on the respondent’s memory but they are usually associated with a higher respondent burden compared to the retrospective methods [3]. In order to reduce the respondent burden during the research, a smaller number of days covered by the prospective method may be considered. 

There are several semi-quantitative FFQs developed to assess the intake of one nutrient and validated against other dietary assessment methods among young adults in Poland [6,7,8,9]. For example, the FFQ developed to assess calcium intake (ADOS-Ca) was validated against the repeated 24 h dietary recall among university students, both men and women [6]. Some other FFQs have also been developed to evaluate the intake of single nutrients, for example: vitamin D [7], zinc [8], and magnesium [9], and they were validated against the 3-day dietary record among young females. However, there are only a few semi-quantitative FFQs allowing the evaluation of the habitual diet as a whole [10,11,12,13]. One of them was developed to assess the children’s dietary intake and validated against the repeated 24 h dietary recall among 3-year-old children [10]. Two other FFQs have been developed for the assessment of dietary intake in adults [11,13]. Reproducibility and validity of the 134-item FFQ in estimating the energy and nutrient intake were tested among adults living in urban and rural areas of Poland [11]. The 165-item FFQ^®^ was tested for the test–retest reproducibility of the frequency and amount of consumption of 44 food groups among university students [12]. Furthermore, the energy and nutrient intake obtained with the 165-item FFQ^®^ was compared with the results from the 3-day food record among young females [13]. Since the 165-item FFQ^®^ contains a long food list including detailed questions, e.g., on the seasonal consumption of some fruit and vegetables, it is related to a high respondent burden. Thus, to conduct nutritional epidemiological studies in large samples of adults in Poland, a validated semi-quantitative FFQ with a shorter food list and a simpler way to analyze the crude data is needed. 

The 72-item semi-quantitative food frequency questionnaire (72-item SQ-FFQ) was developed to estimate the habitual food consumption frequency as well as energy and nutrient intake, based on a non-quantitative FFQ covering 62 food items (62-item FFQ-6®) [14]. The 62-item FFQ-6^®^ was previously tested for the test–retest reproducibility and identifying dietary patterns among 13–21-year-old females. When developing the 72-item SQ-FFQ, the food list of the 62-item FFQ-6^®^ has been reasonably extended and modified to better capture the energy and nutrient intake. Therefore, the aim of this study was to assess the reproducibility and relative validity of the 72-item semi-quantitative food frequency questionnaire (72-item SQ-FFQ) among Polish young adults, both males and females. 

## 2. Materials and Methods

### 2.1. Study Design and Participants

This study was conducted in the years 2014–2016 as part of a larger survey. Potential participants were informed about the aims, scope, and organization of the study. Written informed consent was obtained from all people who agreed to participate in the study. The study included two meetings with each respondent. At the first meeting, respondents were asked to fill in the self-administered questionnaires in a paper-and-pencil form, including the 72-item SQ-FFQ (test) and the set of questions on sociodemographics and lifestyle, as well as to prepare the 2-day estimated food record (FR) at home for the next meeting. The well-trained researchers instructed respondents in detail on how to complete the questionnaires and the FR and replied to any doubts. Anthropometric measurements (body height and weight) were conducted during the meeting by the researchers, according to the international guidelines [15,16]. At the second meeting (after two weeks), respondents provided the FR and completed the 72-item SQ-FFQ again (retest). The researchers answered comprehensively any questions regarding the study. 

The study was carried out on a convenience sample of Polish students attending the University of Warmia and Mazury in Olsztyn, Poland. Students from 19 to 26 years old, from different faculties and years of study, were invited to participate in the research. Initially, 202 respondents participated in the study, of which 12 were excluded due to the incompleteness of self-reported data and 4 were excluded due to implausible energy intake obtained with FR. Finally, the data from 186 university students (47.8% of females) were analyzed.

### 2.2. The 72-Item Semi-Quantitative Food Frequency Questionnaire (72-Item SQ-FFQ)

The 72-item SQ-FFQ is a semi-quantitative FFQ assessing the consumption of 72 food items over the past year. The 72-item SQ-FFQ was developed to assess the habitual dietary intake of adults, based on a non-quantitative food frequency questionnaire covering 62 food items (62-item FFQ-6®) [14]. The 62-item FFQ-6^®^ was developed by Lidia Wadolowska and Ewa Niedzwiedzka. The authors of the present study made a further modification to the questionnaire to obtain a new semi-quantitative version. The 62-item FFQ-6^®^ has been supplemented with 10 food items to better capture the energy and nutritional value of a habitual diet, and finally included 72 food items. For example, fast foods (e.g., pizza, hamburgers) and flour foods (e.g., dumplings, pancakes) have been added to the FFQ list. To allow a more accurate dietary assessment in the context of diet-related diseases, some of the original food items have been divided into pro-health and less healthy foods, e.g., the food item ‘Nuts and nut spreads’ (the 62-item FFQ-6®, question no. 50) was divided into two food items: ‘Nuts’ and ‘Nut spreads’ (the 72-item SQ-FFQ, questions no. 54 and 55; Table S1). 

The questionnaire contains two questions on the total consumption frequency of fruit and vegetables (questions no. 31 and 42; Table S1) and fifteen single items of fruit (8 questions: no. 32–39) and vegetables (7 questions: no. 43–49). The two questions assessing the total consumption frequency of fruit and vegetables can be applied by researchers without considering single items, thus these questions allow to reduce the length of the questionnaire. Secondly, these two questions can be used to adjust the consumption frequency of fruit and vegetables obtained using fifteen single items when the full version of the questionnaire was used. Such an approach allows minimizing the possible overestimation of the consumption due to many separate questions about different types of fruit and vegetables included in the FFQ [5]. In the present study, the adjustment of consumption frequency of single items of fruit and vegetables was applied according to the manual presented in the previous study [14].

Each respondent reported habitual frequency of consumption of the food items by indicating one of the six categories, which were converted into daily frequency (times/day): never or almost never (0), once a month or less (9/365 = 0.025), several times a month (3/30 = 0.1), several times a week (4/7 = 0.571), daily (1), several times a day (2). To convert the data, the same daily frequencies were used as established for the 62-item FFQ-6^®^ [14], which were calculated based on the literature review [17] and the previous experiences of Wadolowska’s research team [6,12,13].

Initially, the modified FFQ (72-item SQ-FFQ) included the part regarding the evaluation of consumed portion size in three categories: small, medium, and large, according to the album of photographs of food products and dishes [18]. However, the relative validity results obtained for the FFQ with separate portion-size questions were not significantly better than those obtained using standard portion sizes adopted for each food item. The difference in relative validity correlations between both approaches ranged from −0.03 to 0.07 for the analyzed dietary components. Therefore, the standard portions were applied to estimate the dietary intake from the 72-item SQ-FFQ. Such an approach made the 72-item SQ-FFQ easier to use by both respondents and researchers. The standard portion sizes have been adopted for the representative of each food item based on the medium portion shown in the Polish album of photographs of food products and dishes [18] as well as the data on food portion sizes obtained in other countries [19,20,21]. The adopted standard portion sizes are universal for Polish adults, both males and females. The list of 72 food items, their representatives, and adopted standard portion sizes are presented in Table S1. 

To estimate the dietary intake, two questions on the total consumption frequency of fruit and vegetables (used to adjust the consumption frequency of single items of fruit and vegetables) were excluded from the analysis to avoid duplicate data. Thus, 70 food items were included. The daily frequency of food consumption (times/day) was multiplied by the standard portion size of the representative of the food item. Next, the mean daily energy and nutrient intake obtained with the 72-item SQ-FFQ was calculated for each respondent using Polish food composition tables [22]. For food items that were not available in the Polish tables (e.g., venison), the online food composition database developed by the United States Department of Agriculture (USDA) was used [23].

### 2.3. The Estimated Food Record (FR)

The estimated food record (FR) was used as a reference method to validate the 72-item SQ-FFQ. The FR covered two non-consecutive days (the days were chosen from all days of the week in different combinations). Participants recorded the type and amount of consumed foods, dishes, and beverages at the time of consumption. The consumed portions of food were recorded in household measures and then expressed in grams [3]. To assess the food portion size, the album of photographs of food products and dishes [18] was used as an aid.

Daily energy and nutrient intake were calculated for each respondent using the computer program DIETA 6 (National Food and Nutrition Institute, Warsaw, Poland), including Polish food composition tables [22]. The intake of energy, 38 nutrients, and alcohol was analyzed. Dietary supplementation was not taken into account. 

Regarding data cleaning, respondents’ mean daily energy intake obtained with FR was considered biologically implausible if it was lower than 800 kcal or higher than 4200 kcal among males, and lower than 600 kcal or higher than 3500 kcal among females [24]. 

### 2.4. Other Variables

Sociodemographic and lifestyle data were collected as self-reports, using closed questions except for age (calculated from the date of the research and the date of birth). Six sociodemographic variables such as: sex, age (in years), place of residence (3 categories), mother’s education (3 categories), father’s education (3 categories), family economic situation (3 categories), and three lifestyle variables such as physical activity (PA) at work/school (3 categories), PA at leisure time (3 categories), screen time (3 categories) were analyzed to characterize the participants. PA was assessed using two questions with a brief description of each response category, including activity duration and examples [25]. 

Anthropometric measurements were conducted by well-trained researchers according to the international guidelines [15,16]. Body height was measured using mobile stadiometers (SECA), and body weight was measured using digital scales (Tanita). Participants were measured in light clothes, without shoes. Thus, corrections for body weight were used (0.5 to 1.0 kg). BMI (kg/m^2^) was calculated as the body weight divided by the square of body height (in meters). Respondents were classified into four BMI categories: underweight (<18.5 kg/m^2^), normal weight (18.5–24.9 kg/m^2^), overweight (25.0–29.9 kg/m^2^), and obesity (≥30.0 kg/m^2^) [15].

### 2.5. Statistical Analysis

Normality was tested using the Kolmogorov–Smirnov test. The means and standard deviations (SD) for continuous variables and percentage distributions for categorical variables were calculated. For sample characteristics, the means were compared between sexes using the Mann–Whitney test (for two independent samples), and proportions were compared using the chi-squared test. Since the proportion of male and female subgroups was similar, further analyses were performed for the total sample of young adults. 

The intakes of energy, 38 nutrients, and alcohol (dietary components) obtained from both administrations of the 72-item SQ-FFQ (FFQcrude and FFQretest) as well as both dietary assessment methods (FFQ and FR) were compared. The energy intake obtained with both methods (FFQcrude, FRcrude) was standardized to 2000 kcal/day (FFQstand, FRstand) for comparison of the nutrient content in the diet irrespective of the energy intake (energy-adjusted data). The dietary intake obtained with FFQcrude was adjusted using linear regression analysis (FFQreg) to obtain the results as close as possible to the dietary intake obtained with the FRcrude as the reference method (regression-adjusted data). The regression equations for energy, nutrient, and alcohol intake obtained with the 72-item SQ-FFQ are presented in Table S2. 

The test–retest reproducibility and relative validity of the 72-item SQ-FFQ were assessed on: (i) the group level—(1) comparing the mean values of intakes of dietary components using the Wilcoxon signed-rank test (for two dependent samples), (2) calculating the relative difference (RD) between mean values of intakes of dietary components obtained with FFQ compared to the reference method (FRcrude or FFQretest), and expressed as a percentage (%); (ii) the individual level by: (1) Spearman’s correlation coefficient, (2) intraclass correlation coefficient (ICC)—to assess the test–retest reproducibility, and (3) cross-classification analysis (quartiles of intake). Since the use of correlation can be misleading, the Bland–Altman method was applied to assess agreement between two methods (FFQ and FR) as well as between both administrations of the FFQ [26]. The Bland–Altman method can be interpreted at both group and individual levels. Regarding the Bland–Altman method, mean intake from both methods, mean difference in intake between both methods, 95% limits of agreement (LOA) as ±1.96 SD of the mean difference, and the Bland–Altman index (B-A index) defined as a percentage of respondents beyond LOA were calculated. Since it is recommended that min. 95% of differences should be within the LOA, the B-A index amounting max. 5% indicates a good agreement between the methods [26,27]. The strength of correlation was interpreted as follows: 0.01–0.09 negligible, 0.10–0.29 weak, 0.30–0.49 moderate, 0.50–0.69 strong, 0.70–0.89 very strong, 0.90–1.00 perfect [28]. The ICC was interpreted as follows: <0.50 poor reliability, 0.50–0.74 moderate, 0.75–0.90 good, >0.90 excellent [29]. While data on the cross-classification analysis of nutrient intake were interpreted, the recommended proportions of correctly classified respondents above 50% and grossly misclassified respondents below 10% were considered [30]. Cross-classification analysis between both methods (FFQ and FR) was not performed for alcohol intake due to the specific distribution of alcohol intake obtained with the FR (24.2% of respondents reported alcohol consumption, thus they fell into one quartile).

The statistical analysis was performed using STATISTICA 13 (Dell Inc.; Tulsa, OK, USA; StatSoft Polska, Cracow, Poland) and PS IMAGO PRO 7.0 on an IBM SPSS Statistics 27 analytical engine (Predictive Solutions, Cracow, Poland). *p*-values < 0.05 were considered statistically significant.

## 3. Results

Characteristics of the participants regarding: age, place of residence, mother’s education, father’s education, family economic situation, PA at work/school, PA at leisure time, screen time, and body weight status assessed in four BMI categories are presented in Table 1. There were significant differences between sexes for six out of nine characteristics. The mean age of males was slightly higher than females (21.7 vs. 21.1 years). Although the proportions of participants living in villages, towns, and cities in the total sample were similar, there was a higher percentage of males than females living in the villages (42.3% vs. 22.5%). Compared with females, a higher percentage of males assessed their family economic situation as above average (39.2% vs. 14.6%), their physical activity at work/school as high (15.5% vs. 4.5%), and their screen time as at least 4 h/day (19.6% vs. 6.7%). The proportion of males with overweight was 3 times higher than females (34.0% vs. 11.3%), and over 9 times more males than females were obese (10.3% vs. 1.1%).

### 3.1. Reproducibility of the 72-Item SQ-FFQ

The significant differences in the mean daily intake obtained from both administrations of the 72-item SQ-FFQ were found for energy and 26 out of 39 dietary components (Table 2). The mean daily energy and dietary component intake obtained with FFQcrude was overestimated compared to FFQretest, except for insignificantly underestimated eicosapentaenoic acid (EPA), docosahexaenoic acid (DHA), and alcohol. The mean daily energy intake was 1992 kcal and 1912 kcal, respectively (*p* = 0.003). The RD for energy intake was 4.2%, and for dietary component intake, it ranged from −2.9% for DHA to 8.4% for calcium. 

The Spearman’s correlations and ICC between FFQcrude and FFQretest were significant for the intake of energy and all dietary components (Table 3). The Spearman’s correlation coefficient was 0.878 for energy intake, and for dietary component intake, it ranged from 0.631 for β-carotene to 0.876 for protein. The ICC was 0.917 for energy intake and ranged from 0.583 for β-carotene to 0.935 for monounsaturated fatty acids (MUFA). 

The cross-classification analysis showed that 69.4% of the respondents were classified into the same quartiles of energy intake obtained with FFQcrude and FFQretest, 95.7% into the same or adjacent quartiles, and none of the respondents were classified into extreme quartiles (Table 4). The percentage of respondents classified into the same quartiles of dietary component intake ranged from 57.0% for vitamin A to 74.2% for alcohol, into the same or adjacent quartiles ranged from 84.9% for β-carotene to 97.8% for protein, and into the extreme quartiles ranged from 0.0% for 17 dietary components to 2.7% for β-carotene. 

**Figure 1:**
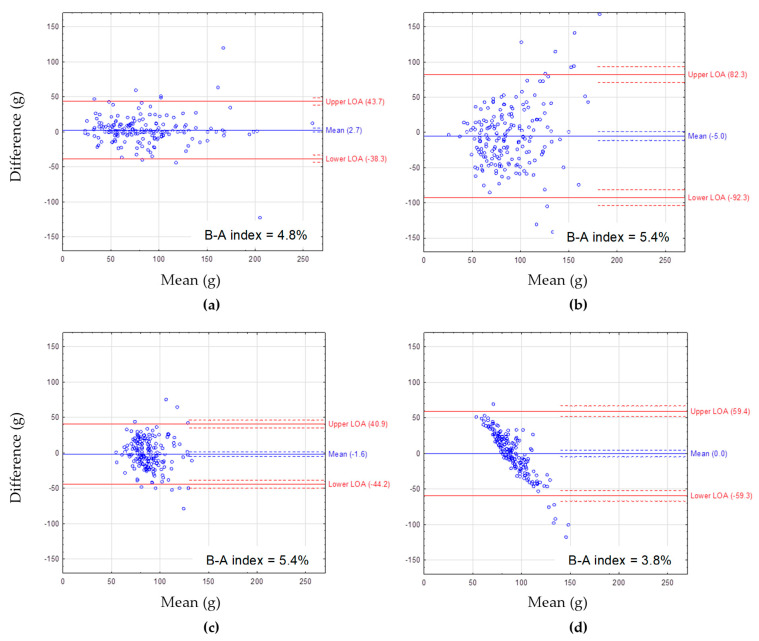
The Bland–Altman plots for protein intake assessed with: (**a**) FFQcrude and FFQretest, (**b**) FFQcrude and FRcrude, (**c**) FFQstand and FRstand, (**d**) FFQreg and FRcrude. FFQcrude—crude data from the first administration of the FFQ (test), FFQretest—crude data from the second administration of the FFQ, FRcrude—crude data from the 2-day estimated food record (FR), FRstand—the FR data after standardization of energy intake, FFQstand—the FFQ data after standardization of energy intake, FFQreg—the FFQ data adjusted by regression. Mean—mean difference in protein intake between the methods (blue solid line) with 95% CI (dashed lines). LOA—95% limits of agreement between the methods (red solid lines) with 95% CI (dashed lines). B-A index—the Bland–Altman index calculated as percentage of respondents beyond LOA.

**Figure 2:**
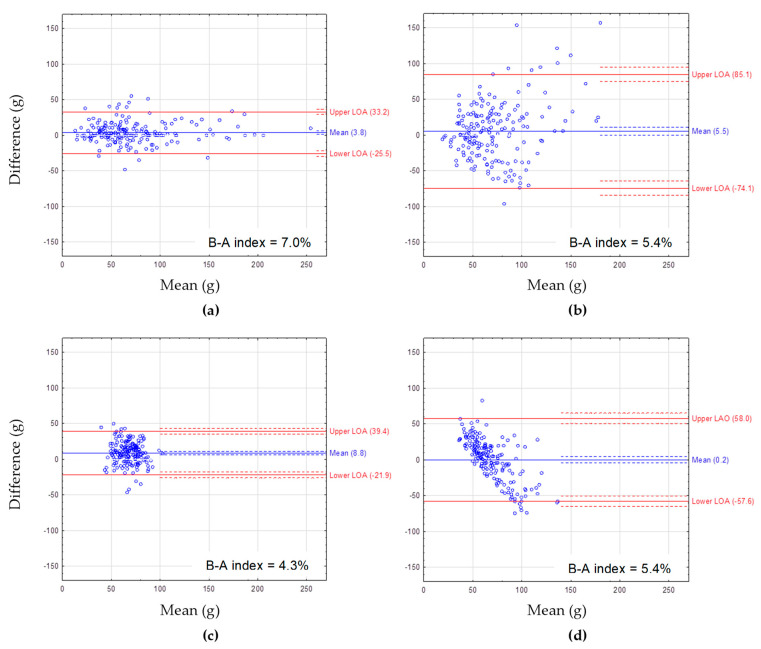
The Bland–Altman plots for fat intake assessed with: (**a**) FFQcrude and FFQretest, (**b**) FFQcrude and FRcrude, (**c**) FFQstand and FRstand, (**d**) FFQreg and FRcrude. FFQcrude—crude data from the first administration of the FFQ (test), FFQretest—crude data from the second administration of the FFQ, FRcrude—crude data from the 2-day estimated food record (FR), FRstand—the FR data after standardization of energy intake, FFQstand—the FFQ data after standardization of energy intake, FFQreg—the FFQ data adjusted by regression. Mean—mean difference in fat intake between the methods (blue solid line) with 95% CI (dashed lines). LOA—95% limits of agreement between the methods (red solid lines) with 95% CI (dashed lines). B-A index—the Bland–Altman index calculated as percentage of respondents beyond LOA.

**Figure 3:**
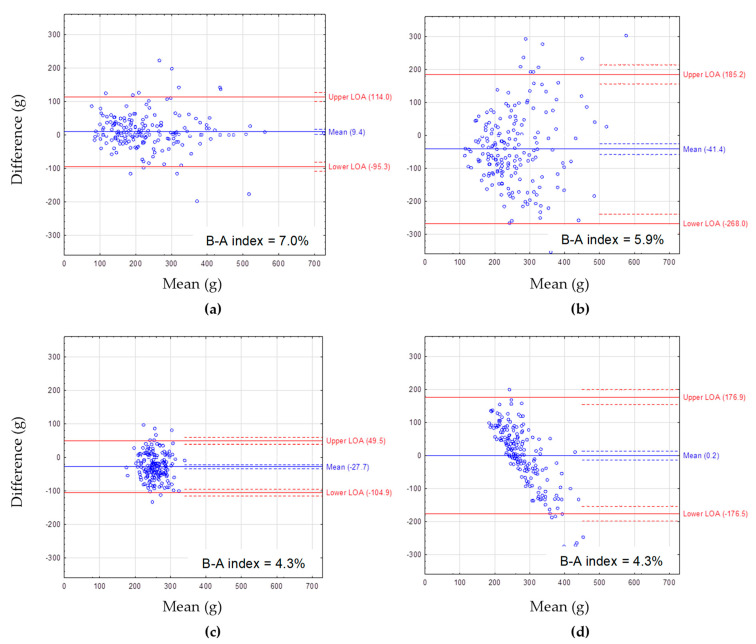
The Bland–Altman plots for carbohydrate intake assessed with: (**a**) FFQcrude and FFQretest, (**b**) FFQcrude and FRcrude, (**c**) FFQstand and FRstand, (**d**) FFQreg and FRcrude. FFQcrude—crude data from the first administration of the FFQ (test), FFQretest—crude data from the second administration of the FFQ, FRcrude—crude data from the 2-day estimated food record (FR), FRstand—the FR data after standardization of energy intake, FFQstand—the FFQ data after standardization of energy intake, FFQreg—the FFQ data adjusted by regression. Mean—mean difference in carbohydrate intake between the methods (blue solid line) with 95% CI (dashed lines). LOA—95% limits of agreement between the methods (red solid lines) with 95% CI (dashed lines). B-A index—the Bland–Altman index calculated as percentage of respondents beyond LOA.

The Bland–Altman plots showing the agreement between FFQcrude and FFQretest are presented for macronutrient intake: protein (Figure 1), fat (Figure 2), and carbohydrates (Figure 3). The mean difference between methods showed a slight overestimation of macronutrient intake by FFQcrude compared to FFQretest (by 2.7 g/day for protein, 3.8 g/day for fat, 9.4 g/day for carbohydrate intake). The B-A index amounted to 4.8%, 7.0%, and 7.0%, respectively. The LOA was the narrowest for fat intake. 

### 3.2. Relative Validity of the 72-Item SQ-FFQ

The significant differences in the mean daily intake obtained with FFQcrude and FRcrude were found for energy and 29 out of 39 dietary components, between FFQstand and FRstand for 31 out of 39 dietary components, and between FFQreg and FRcrude for only 9 out of 39 dietary components: α-linolenic acid (ALA), EPA, DHA, vitamin A, retinol, β-carotene, vitamin D, vitamin B12, alcohol (Table 2). The mean daily intake was significantly underestimated by FFQcrude compared to FRcrude for energy and 22 dietary components, for 18 dietary components by FFQstand vs. FRstand, and slightly underestimated for 5 dietary components by FFQreg vs. FRcrude. For example, the mean daily energy intake from FFQcrude was lower than from FRcrude (1992 kcal vs. 2094 kcal, respectively; *p* = 0.025). After adjustment of the dietary intake using linear regression analysis, the mean daily energy intake from FFQreg compared to FRcrude was similar (2090 kcal vs. 2094 kcal, respectively; *p* = 0.510). The RD for FFQcrude vs. FRcrude was −4.9% for energy intake, while for dietary component intake it ranged from −50.5% for fructose to 94.7% for DHA. The RD for FFQstand vs. FRstand ranged from −44.7% for sodium to 138.3% for alcohol. The RD for FFQreg vs. FRcrude was −0.2% for energy intake, and for dietary component intake, it ranged from −0.8% for alcohol to 0.6% for EPA. 

The Spearman’s correlation between FFQcrude and FRcrude was 0.353 for energy intake, and for dietary component intake, it ranged from −0.025 for iodine to 0.390 for MUFA. The correlations for the energy and dietary component intake were significant, except for 5 out of 39 dietary components (i.e., EPA, DHA, calcium, iodine, vitamin A) (Table 3). The correlations between FFQstand and FRstand ranged from 0.021 for iodine to 0.546 for manganese. All correlations were significant, except for 9 out of 39 dietary components (i.e., polyunsaturated fatty acids (PUFA), linoleic acid (LA), ALA, EPA, DHA, sodium, iodine, niacin, vitamin B6). The correlation between FFQreg and FRcrude was 0.353 for energy intake, and for dietary component intake, it ranged from 0.028 for iodine to 0.391 for MUFA. All correlations were significant, except for 4 out of 39 dietary components (i.e., EPA, DHA, calcium, vitamin A). 

The cross-classification of the energy intake obtained with FFQcrude and FRcrude showed that 35.5% of the respondents were classified into the same quartiles, 73.1% into the same or adjacent quartiles, and 5.9% into extreme quartiles (Table 4). Similar results were found for FFQreg and FRcrude—it was 35.5%, 73.7%, and 5.9% of the respondents, respectively. The percentage of respondents classified into the same quartiles of dietary component intake obtained with FFQcrude and FRcrude ranged from 23.1% for iodine to 40.3% for niacin, for FFQstand and FRstand ranged from 24.7% for both ALA and EPA to 43.5% for phosphorus, and for FFQreg and FRcrude ranged from 22.6% for iodine to 39.8% for niacin. The proportion of respondents classified into the same or adjacent quartiles for FFQcrude and FRcrude ranged from 61.3% for EPA to 76.9% for niacin, for FFQstand and FRstand ranged from 63.4% for EPA to 83.9% for manganese, and for FFQreg and FRcrude ranged from 60.2% for iodine to 76.9% for niacin. The percentage of respondents classified into the extreme quartiles for FFQcrude and FRcrude ranged from 3.2% for plant protein to 11.8% for iodine, for FFQstand and FRstand ranged from 1.6% for both magnesium and manganese to 14.0% for both sodium and vitamin B6, and for FFQreg and FRcrude ranged from 3.8% for plant protein to 10.8% for both EPA and vitamin A. 

The Bland–Altman plots showing the agreement between the 72-item SQ-FFQ and FR are presented for macronutrient intake (Figure 1, Figure 2 and Figure 3). For protein intake, the mean difference between methods ranged from 0.0 g/day for FFQreg and FRcrude to −5.0 g/day for FFQcrude and FRcrude (Figure 1). For fat intake, the mean difference between methods ranged from 0.2 g/day for FFQreg and FRcrude to 8.8 g/day for FFQstand and FRstand (Figure 2). For carbohydrate intake, the mean difference between methods ranged from 0.2 g/day for FFQreg and FRcrude to −41.4 g/day for FFQcrude and FRcrude (Figure 3). Mean differences between methods showed underestimation of protein and carbohydrate intake by FFQcrude vs. FRcrude and FFQstand vs. FRstand. The LOA was the narrowest for FFQstand and FRstand and the widest for FFQcrude and FRcrude, regardless of the macronutrient. The B-A index ranged from 3.8% for protein intake from FFQreg and FRcrude to 5.9% for carbohydrate intake from FFQcrude and FRcrude.

## 4. Discussion

### 4.1. Reproducibility

The test–retest reproducibility of the 72-item SQ-FFQ was good at both an individual and group level. 

On the group level, the energy and nutrient intake was slightly overestimated by the test compared to the retest of the 72-item SQ-FFQ, except for EPA, DHA, and alcohol which were insignificantly underestimated. The relative differences in dietary intake obtained with test and retest were small (not exceeding ±10%), although they were found for energy and two-thirds of the analyzed dietary components. The overestimation of the absolute energy and nutrient intake obtained from the first administration of the FFQ compared to the second one was found in other studies conducted among adults from different countries of the world [11,31,32,33,34,35]. 

As recommended [30], more than 50% of the respondents were classified into the same quartiles of intake of all analyzed dietary components (57.0–74.2%) estimated in both administrations of the 72-item SQ-FFQ, and the gross misclassification was very low (0–2.7%). These reproducibility findings were somewhat similar to those obtained in Switzerland (same tertiles: 40.0–77.5%, extreme: 0–10.0%) [32], New Zealand (same tertiles: 46.9–79.6%, extreme: 1.4–6.1%) [34], China (same tertiles: 57.8–71.4%, extreme: 1.3–7.8%) [35], and better than obtained for the FFQ validated in Canada (same quartiles: 34.4–62.5%) [31].

The Spearman’s correlations between both administrations of the 72-item SQ-FFQ were strong to perfect (r = 0.631–0.878), and intraclass correlations were moderate to excellent (ICC = 0.583–0.935). According to the literature review, common correlations between two administrations of an FFQ are from 0.5 to 0.7 [4]. In the present study, the test–retest correlations for the vast majority of dietary components (38 out of 40) were higher than 0.7, with the highest value for energy intake (r = 0.878, ICC = 0.917). For example, the reproducibility of energy intake estimated by the FFQ was lower in other research carried out in Poland (r = 0.46, ICC = 0.40 for rural residents; r = 0.43, ICC = 0.42 for urban residents) [11], Spain (r = 0.66, ICC = 0.79) [36], Switzerland (ICC = 0.72) [32], and Canada (r = 0.73) [31], while higher among adults from Lebanon (ICC = 0.945) [33]. According to the recent literature review on FFQs administered to adults from various countries, reproducibility correlations for crude energy and nutrient intake varied from 0.16 to 0.96 (ICC: 0.10–0.998) [37]. The review of FFQs validated in Japan showed that median correlations between two FFQs for nutrient intake ranged from 0.24 to 0.74 [38]. High correlations obtained in the present study may be partially explained by the relatively short time interval between two administrations of the FFQ (two weeks). The literature review showed higher correlations for repeated administrations of the FFQ with an interval of 1 month or less compared to the longer period of time, e.g., 6 months to 1 year (for energy: 0.68 vs. 0.60, respectively) [4]. On the other hand, the differences between the test and the retest administered after a long period of time may not reflect measurement error, but actual changes in habitual diet. 

Since the standard food portions were applied to compute dietary intake from the 72-item SQ-FFQ, the test–retest differences depended on the assessment of the frequency of food consumption. On the individual level, the test–retest reproducibility was the lowest for β-carotene, fructose, and vitamin C which may indicate the respondents’ difficulties in assessing the consumption frequency of fruit and vegetables. For these food groups, several separate questions were included in the 72-item SQ-FFQ. This can lead to an overestimation of fruit and vegetable consumption [5]. In the present study, to minimize the bias, the consumption frequencies of individual fruit and vegetable items were adjusted by the total consumption of fruit and vegetables (two food items). 

### 4.2. Relative Validity

The relative validity of the 72-item SQ-FFQ against the FR was acceptable. 

On the group level, the crude energy intake was underestimated by 103 kcal/day (4.9%) with the 72-item SQ-FFQ compared to the 2-day FR (2094 kcal/day). A significant underestimation of intake by the FFQ was found for more than half of the analyzed dietary components (56%, crude data), while an overestimation of intake was observed for fatty acids, alcohol, and vitamin B12 estimated from both crude and energy-adjusted data. As in the present study, an underestimation of energy intake by the FFQ was found among older adults from New Zealand (by 7.3%) [34], but several other FFQs significantly overestimated the energy intake compared to the reference methods [13,30,39,40]. The findings obtained for the previously developed Polish FFQ (the 165-item FFQ®) showed that the intake of energy and most of the analyzed nutrients was substantially overestimated by the FFQ [13], which could have been due to the much longer food list included in the 165-item FFQ^®^ than in the 72-item SQ-FFQ. 

Considering macronutrients, the 72-item SQ-FFQ underestimated the absolute intakes of total protein and carbohydrates, and overestimated fat intake based on crude and energy-adjusted data. Therefore, taking into account the relatively small level of underestimation of total protein intake, the underestimation of energy intake resulted from the underestimation of the carbohydrate intake. Similar to our findings, an overestimation of intake of fat and some fatty acids by the FFQ was found among Croatian men and women (fat, SFA, MUFA) [39], Scottish men and women [30], Canadian women (fat, MUFA) [31], and Mexican adults (fat, SFA, MUFA) [40]. However, contrary to our results, the intakes of total protein and carbohydrates were overestimated in some of the above-mentioned studies as well [30,39]. 

Dietary components such as fiber, folates, vitamin C, β-carotene, and fructose were underestimated by the FFQ (generally from −18% to 50%), which may have resulted from the underestimation of fruit and vegetable intake by the FFQ. The use of weights adjusted the consumption frequency of fruit and vegetables obtained with multiple separate questions to the consumption frequency reported in two general questions. The weights were applied to reduce the possible overestimation of the fruit and vegetable consumption due to many separate questions about their different types in the FFQ list [5]. However, two single questions assessing the overall consumption of fruit and vegetables seem to underestimate their intake compared to the FR method. The previously developed Polish FFQ (the 165-item FFQ®) overestimated the intake of fiber, folic acid, vitamin C, and β-carotene, which could have resulted from a long food list with detailed questions on the seasonal consumption of fruit and vegetables [13]. Besides the structure of a questionnaire, fruit and vegetables are considered ‘very healthy’ food and their consumption can be overestimated due to social approval bias [41].

Regarding the individual level, the correlations between the 72-item SQ-FFQ and FR were negligible to moderate for crude dietary intakes (−0.025–0.390), while negligible to strong after energy adjustment (0.021–0.546). Although the findings from other studies are difficult to compare due to differences in the structure and validation of FFQs, the relative validity correlations (crude data) were similar for the FFQ validated among adults from Croatia (r = 0.098–0.482) [39] and other FFQ validated in Poland (rural residents: 0.20–0.48, urban residents: 0.10–0.51) [11]. Somewhat higher correlations between FFQs and reference methods were found among adults from Portugal (0.21–0.73) [42], Lebanon (0.16–0.65) [33], and older adults from New Zealand (0.12–0.78) [34]. According to the recent literature review on FFQs administered to adults from various countries, validity correlations for crude energy and nutrient intake varied from −0.38 to 0.998 [37].

The ability of the 72-item SQ-FFQ to rank individuals into categories of the dietary intake was similar to that of other FFQ validated in Canada (same quartiles: 26.8–47.9%) [31], while somewhat lower than in Switzerland (same tertiles: 27.5–67.5%, extreme: 7.5–22.5%) [32], New Zealand (same tertiles: 31.6–68.0%, extreme: 2.7–18.4%) [34], and clearly lower than obtained for other FFQ validated in Poland (same quartiles: 67.1–78.1% for rural residents, 65.8–87.7% for urban residents) [11] and Lebanon (same quartiles: 49.2–70.6%, extreme: 3.7–12.2%) [33]. 

Considering the energy intake only, the validity correlation was 0.353 and the exact agreement across quartiles was 35.5% in the present study. According to several literature reviews, the correlations for energy intake between FFQs and reference methods were in the range −0.14–0.92 [4], 0.16–0.77 [43], 0.20–0.87 [38], and 0.11–0.998 (crude data) [37], with median correlations slightly higher (0.46) [4,38] compared to the present study. However, the relative validity of crude energy intake obtained with the 72-item SQ-FFQ was somewhat similar to the results demonstrated for the FFQs validated among adults in Germany (predefined portion size: r = 0.32 for men, r = 0.22 for women) [44], Scotland (men: r = 0.24, agreement: 34%; women: r = 0.39, agreement: 58%) [30], Canada (women only: r = 0.29, agreement: 39.4%) [31], Switzerland (r = 0.31, agreement: 47.5%) [32], Croatia (r = 0.336 (adjusted to sex)) [39], and New Zealand (r = 0.37, agreement: 43.5%) [34]. 

In the present study, the lowest and statistically insignificant correlations between both methods were found for iodine, EPA, vitamin A, calcium, and DHA, while the highest for MUFA, fat, energy, and plant protein (crude data). The cross-classification analysis confirmed the lowest ability of the 72-item SQ-FFQ to rank respondents for the intakes of iodine and EPA. The low relative validity of the 72-item SQ-FFQ for iodine, EPA, and DHA can be explained by the rare consumption of fish by respondents (lean fish: 0.14 times/day, oily fish: 0.09 times/day; approx. several times a month), and thus difficulties in capturing usual consumption of fish with the FR. Seafood is a good source of iodine as well, but rarely eaten by Poles and therefore was not included in the FFQ list. This may partly explain the high test–retest reproducibility of iodine intake but low relative validity. Both sodium and iodine intake were underestimated by FFQ (by 47% and 45% for crude data, respectively). Similar results were obtained for energy-adjusted data. Iodization of salt is obligatory in Poland [45]. The salt given in the recipes of the dishes was covered by both methods, but the salting of meals at the table was not included in the FFQ. 

In contrast to the high reproducibility of alcohol intake (r = 0.856, ICC = 0.854; 74.2% of respondents correctly classified) estimated by the 72-item SQ-FFQ, the relative validity of alcohol intake was weak (r = 0.274), and no improvement was observed after data adjustment. According to the literature, the relative validity of alcohol intake estimated with the FFQs is usually high compared to other dietary components [38,43]. The high inter-individual variability in alcohol consumption should lower the relative validity. However, some people consume a lot of alcoholic beverages, and others not at all, which may facilitate the reporting of alcohol intake and increases the validation results [38]. In the present study, the FR covered two non-consecutive days which seems to be insufficient to capture alcohol consumption adequately. Only 24.2% of respondents reported alcohol intake in the FR.

According to the meta-analysis of FFQ validation studies, the correlations between FFQs and reference methods ranged from 0.45 (energy, protein) to 0.74 (alcohol) [43]. Another review showed the lowest correlations for vitamin A (0.39), while the highest for fat (0.51) and calcium (0.55) [4]. The review of FFQs validated against diet records in Japan showed the lowest median correlation for niacin (0.23), PUFA (0.29), sodium or NaCl (0.33), and vitamin A (0.35), while the highest for fiber (0.56), SFA (0.57), and calcium (0.58) [38]. Considering the ten most frequently mentioned nutrients in the FFQ validation studies (crude data), the lowest correlation was found for PUFA (−0.10) and the highest for vitamin C (0.987) [37].

The differences in dietary intake estimates between the FFQ and the reference method could result from the high variability in intake of some nutrients (e.g., vitamin A) [46,47], as well as the researchers’ decisions regarding the estimation of the energy and nutrient intake based on the FFQ data, i.e., the choice of representatives for the food items, standard food portions, and the short-term reference method used. In the present study, standard food portions, universal for both men and women, were applied. They were adopted mainly based on the medium portions in the Polish album of photographs of food products and dishes [18]. Therefore, they may be inappropriate for certain groups of people, for example, overweight/obese or those with high physical activity. Thus, a relatively high proportion of overweight/obese men in the present study may have biased the findings to some extent. In general, the use of standard portions decreases inter-individual variability and can lead to an error in estimating absolute intake [24,43]. For example, a tendency to underestimate the nutrient intakes among men and to overestimate among women was shown for the Willet FFQ (without portion-size questions), from which the dietary intake was computed using the standard portions, identical for both sexes [24]. A German study showed that using fitted sex-specific portion sizes, compared to predefined portions identical for both sexes, improved the validity of quantitative estimates of dietary intake from the FFQ but did not clearly correlate with the reference method [44]. The meta-analysis of FFQ validation studies demonstrated higher energy-adjusted correlations for some nutrient intakes (e.g., protein, vitamin C) estimated by FFQs with standard portions compared to those with separate portion-size questions [43]. 

The adjustment of energy and nutrient intake using linear regression analysis improved the agreement between the 72-item SQ-FFQ and FR at the group level, but not at the individual level. The ranges of correlation coefficient and proportion of respondents classified into the same quartiles were similar for crude and regression-adjusted data, and somewhat higher for energy-adjusted data. On the group level, the differences between both methods in mean daily intake of all dietary components were small (RD: −0.8 to 0.6%). For example, the regression-adjusted energy intake was underestimated by the FFQ by 4.2 kcal/day (0.2%). Therefore, the application of the regression equations (Table S2) to estimate the dietary intake from the 72-item SQ-FFQ can be useful in research focusing on the group level of the dietary assessment.

Energy adjustment allowed the intakes of dietary components to be compared regardless of differences in energy intake [4]. Standardization of the energy intake noticeably reduced the variability of dietary intake. The Bland–Altman plots showed the narrowest LOAs for all macronutrients estimated from energy-adjusted data. The energy adjustment improved the validity correlations for vitamin A, fiber, calcium, manganese, phosphorus, and magnesium (by ≥0.2) and the ability of the 72-item SQ-FFQ to rank individuals according to quartiles of intake for similar nutrients. However, it should be noted that after energy adjustment the relative validity decreased for some other nutrients, such as: ALA, LA, PUFA, niacin, vitamin B6, and sodium. Thus, the use of energy-adjusted data should be considered especially in studies focusing on the assessment of habitual intake of the above-mentioned dietary components by the 72-item SQ-FFQ. This is of particular importance when assessing fiber and calcium intakes, for which the validity correlations increased by 1.7 and 2.8 times, respectively. Similarly, after the energy adjustment, an increase in the validity correlations for both calcium and fiber intakes was also found in other studies [31,44]. The meta-analysis of FFQ validation studies demonstrated that the energy-adjusted correlations were higher by 0.02–0.08 for all analyzed nutrients, except for vitamin C [43]. After energy adjustment, the correlations between the Willet FFQ (without portion-size questions) and multiple 24 h dietary recalls improved considerably, while for the Block FFQ (with portion-size questions) modestly [24]. 

### 4.3. Strengths and Limitations

Some limitations of this study should be stated. First, the reproducibility and relative validity of the 72-item SQ-FFQ was assessed among a convenience sample of university students. Other groups of adult Poles (e.g., working people, those with a lower education level, elderly) were not included. Therefore, the results should not be directly extended to the general population of adult Poles. Second, the FR covered two non-consecutive days that can be insufficient to adequately assess habitual intake of alcohol and some nutrients, e.g., iodine, vitamin A, EPA, and DHA, due to the high day-to-day variability in the intake of such nutrients [46,47]. The reference method (FR) was not repeated across the year; thus, it probably did not capture the seasonal variability of food consumption. However, two days of the reference method were chosen in other validation studies [40,44,48]. The use of more days of the reference method is associated with a greater respondent burden. Since using self-reported dietary assessment methods can lead to misreporting of energy intake, the sex-specific cut-offs were applied as exclusion criteria based on the literature [24]. Although it is the simplest method to exclude respondents with misreported energy intakes, it may not identify all individuals who under- or overreported their energy intakes. Next, using representatives of food items may be a possible source of bias in the estimation of the dietary intake from the 72-item SQ-FFQ. Furthermore, some foods can be reported in the FR but not included in the FFQ list, particularly rarely consumed or novel foods. Some foods omitted in the FFQ list can be important to explain inter-individual variability [43]. 

There are several strengths of the study that should be highlighted. First, the validation study was performed in a sufficiently large sample [4], with similar proportions of males and females. Given the common respondents’ difficulties in properly estimating the portion size of consumed foods [2], using standard portion sizes for food items made the 72-item SQ-FFQ easier to use by both respondents and researchers. Therefore, this tool can be used in large studies, with the proper proportion of time, cost, and quality. Next, the adjustment for single items of fruit and vegetables was applied to minimize the possible overestimation of their consumption frequency due to many separate questions about different types of fruit and vegetables in the FFQ [5]. Since misreporting of energy intake is a common problem in dietary assessment [4], the respondents with implausibly low or high energy intakes obtained with the FR were excluded from further analysis. The dietary intake was adjusted using two techniques—standardization of energy intake and linear regression analysis. Energy adjustment allows to minimize the bias in the estimation of dietary intake and focus on the diet composition [4,43]. It is worth emphasizing that the results are shown for a wide range of dietary components (39 items). Despite the relative validity of the 72-item SQ-FFQ for certain nutrients being weak and disappointing, knowledge of the findings obtained for many dietary components is crucial for other researchers in deciding to choose an optimal tool tailored to their research aim.

## 5. Conclusions

Our findings showed good reproducibility and acceptable relative validity of the 72-item SQ-FFQ among young adults in Poland. Adjustment of energy and nutrient intake using regression equations can improve the assessment of the dietary intake estimated by the 72-item SQ-FFQ at the group level. The ability of the 72-item SQ-FFQ to rank individuals for nutrient intake increased after the energy intake standardization. This tool can be recommended for assessing dietary intake among Polish adults in large-sample studies. However, the intake of certain nutrients should be interpreted with caution. 

## Figures and Tables

**Table 1:** Sample characteristics.

Variables	Total Sample		Males	Females	*p*
	*n*	%	%	%	
Total sample	186	100.0	52.2	47.8	
Age ^1^ (years)	21.4 ± 1.7 (19.0–25.8)		21.7 ± 1.8 (19.0–25.8)	21.1 ± 1.5 (19.3–25.6)	0.020
Place of residence					<0.001
village	61	32.8	42.3	22.5	
town	67	36.0	38.1	33.7	
city ^2^	58	31.2	19.6	43.8	
Mother’s education					0.564
primary/lower secondary	20	10.8	9.3	12.4	
upper secondary	95	51.1	54.6	47.2	
higher	71	38.2	36.1	40.4	
Father’s education					0.686
primary/lower secondary	34	18.3	20.6	15.7	
upper secondary	102	54.8	53.6	56.2	
higher	50	26.9	25.8	28.1	
Family economic situation					<0.001
below average	8	4.3	4.1	4.5	
average	127	68.3	56.7	80.9	
above average	51	27.4	39.2	14.6	
Physical activity at work/school					0.010
low	85	45.7	37.1	55.1	
moderate	82	44.1	47.4	40.4	
high	19	10.2	15.5	4.5	
Physical activity at leisure time					0.912
low	63	33.9	34.0	33.7	
moderate	65	34.9	36.1	33.7	
high	58	31.2	29.9	32.6	
Screen time (h/day)					<0.001
≤1	74	39.8	27.8	52.8	
2–3	87	46.8	52.6	40.4	
≥4	25	13.4	19.6	6.7	
BMI categories					<0.001
underweight (<18.5 kg/m^2^)	15	8.1	2.1	14.6	
normal weight (18.5–24.9 kg/m^2^)	117	62.9	53.6	73.0	
overweight (25.0–29.9 kg/m^2^)	43	23.1	34.0	11.3	
obesity (≥30.0 kg/m^2^)	11	5.9	10.3	1.1	

^1^: mean and standard deviation; in brackets given minimum-maximum range of age; ^2^: at least 100,000 inhabitants; BMI, body mass index [15]; *p*, significance level of Mann–Whitney test (continuous variable) or chi-squared test (categorical variables) for comparison between sexes.

**Table 2:** Comparison of the mean daily energy and nutrient intake obtained with the 72-item SQ-FFQ and estimated food record (FR) among Polish young adults (*n* = 186).

Nutrient (Unit)	Reproducibility				Relative Validity									
	FFQcrude	FFQretest	RD	*p*	FRcrude	RD	*p*	FFQstand	FRstand	RD	*p*	FFQreg	RD	*p*
	Mean ± SD	Mean ± SD	%		Mean ± SD	%		Mean ± SD	Mean ± SD	%		Mean ± SD	%	
Energy (kcal)	1992 ± 936	1912 ± 949	4.2	0.003	2094 ± 745	−4.9	0.025	2000	2000	0.0	-	2090 ± 290	−0.2	0.510
Total protein (g)	86.0 ± 40.3	83.4 ± 41.0	3.2	0.042	91.0 ± 31.2	−5.5	0.028	87.6 ± 17.0	89.2 ± 19.4	−1.8	0.369	91.0 ± 7.7	0.0	0.419
Animal protein (g)	60.0 ± 30.8	58.2 ± 30.6	3.1	0.098	61.2 ± 25.4	−1.9	0.395	61.1 ± 17.8	60.0 ± 18.9	1.8	0.504	61.1 ± 5.5	−0.1	0.262
Plant protein (g)	25.4 ± 12.3	24.5 ± 12.6	3.9	0.033	28.8 ± 9.8	−11.6	<0.001	25.8 ± 5.3	28.1 ± 5.5	−8.1	<0.001	28.7 ± 3.2	−0.1	0.413
Fat (g)	74.5 ± 41.6	70.6 ± 41.0	5.4	0.001	69.0 ± 32.5	8.0	0.142	72.8 ± 12.6	64.1 ± 14.4	13.7	<0.001	69.2 ± 13.7	0.3	0.367
SFA (g)	27.0 ± 14.4	25.4 ± 14.5	6.3	<0.001	26.2 ± 12.6	3.0	0.835	26.7 ± 5.7	24.5 ± 6.5	9.0	<0.001	26.2 ± 4.9	0.0	0.463
MUFA (g)	30.2 ± 18.3	28.7 ± 17.7	5.2	0.005	26.9 ± 14.2	12.5	0.019	29.2 ± 6.4	24.7 ± 7.5	18.1	<0.001	27.0 ± 6.2	0.5	0.276
PUFA (g)	12.0 ± 7.4	11.5 ± 7.2	4.5	0.026	10.5 ± 5.6	14.9	0.042	11.7 ± 3.7	9.7 ± 3.0	21.3	<0.001	10.4 ± 1.7	−0.5	0.255
LA (g)	9.3 ± 5.8	8.8 ± 5.7	5.2	0.020	8.2 ± 4.5	12.8	0.101	9.1 ± 3.4	7.6 ± 2.4	19.6	<0.001	8.2 ± 1.3	0.1	0.181
ALA (g)	1.9 ± 1.3	1.8 ± 1.2	3.6	0.142	1.7 ± 1.4	12.7	0.003	1.8 ± 0.6	1.5 ± 0.9	19.1	<0.001	1.7 ± 0.3	0.3	0.008
EPA (mg)	128.6 ± 183.3	132.2 ± 196.6	−2.7	0.755	70.9 ± 139.8	81.3	<0.001	126.2 ± 150.4	70.0 ± 146.1	80.2	<0.001	71.4 ± 23.8	0.6	<0.001
DHA (mg)	363.6 ± 539.8	374.5 ± 584.1	−2.9	0.451	186.8 ± 374.3	94.7	<0.001	359.4 ± 447.9	187.6 ± 403.3	91.5	<0.001	186.2 ± 27.0	−0.3	<0.001
Cholesterol (mg)	338 ± 178	316 ± 177	6.9	0.004	355 ± 207	−4.8	0.778	348 ± 152	344 ± 178	1.4	0.396	354 ± 48	−0.4	0.265
Carbohydrates (g)	238.3 ± 106.3	228.9 ± 109.1	4.1	0.010	279.7 ± 96.3	−14.8	<0.001	243.3 ± 32.3	271.0 ± 34.9	−10.2	<0.001	279.9 ± 34.0	0.1	0.378
Fructose (g)	6.3 ± 3.5	6.2 ± 3.6	1.4	0.363	12.8 ± 8.1	−50.5	<0.001	7.1 ± 4.2	12.9 ± 7.8	−44.5	<0.001	12.8 ± 2.1	−0.1	0.282
Sucrose (g)	43.5 ± 29.7	41.8 ± 28.3	4.1	0.073	48.2 ± 31.4	−9.8	0.017	42.5 ± 16.7	45.1 ± 20.6	−5.7	0.089	48.4 ± 9.2	0.2	0.067
Fiber (g)	16.6 ± 6.8	15.9 ± 7.1	4.5	0.049	20.3 ± 7.8	−18.2	<0.001	17.7 ± 5.7	20.5 ± 7.9	−13.8	<0.001	20.4 ± 2.2	0.2	0.129
Sodium (mg)	1777 ± 902	1670 ± 895	6.4	0.001	3344 ± 1330	−46.8	<0.001	1788 ± 390	3231 ± 873	−44.7	<0.001	3338 ± 487	−0.2	0.728
Potassium (mg)	2822 ± 1147	2696 ± 1167	4.7	0.003	3440 ± 1145	−18.0	<0.001	2941 ± 607	3401 ± 796	−13.5	<0.001	3454 ± 275	0.4	0.266
Calcium (mg)	718 ± 338	663 ± 343	8.4	<0.001	813 ± 332	−11.7	<0.001	743 ± 227	814 ± 295	−8.7	0.006	815 ± 51	0.2	0.283
Phosphorus (mg)	1415 ± 600	1354 ± 607	4.5	0.004	1470 ± 473	−3.7	0.083	1464 ± 306	1459 ± 354	0.3	0.741	1472 ± 96	0.2	0.328
Magnesium (mg)	310 ± 131	301 ± 137	3.2	0.048	346 ± 121	−10.4	0.001	324 ± 86	345 ± 101	−5.9	0.004	347 ± 31	0.4	0.257
Iron (mg)	9.7 ± 4.4	9.3 ± 4.4	4.7	0.007	12.6 ± 4.9	−22.6	<0.001	9.9 ± 1.7	12.5 ± 4.7	−20.2	<0.001	12.5 ± 1.2	−0.1	0.072
Zinc (mg)	9.7 ± 4.2	9.3 ± 4.2	5.1	0.011	10.8 ± 3.6	−10.1	<0.001	10.0 ± 1.9	10.7 ± 2.4	−5.8	0.002	10.8 ± 0.9	−0.1	0.605
Copper (mg)	1.09 ± 0.50	1.04 ± 0.52	4.9	0.002	1.27 ± 0.45	−14.6	<0.001	1.13 ± 0.32	1.27 ± 0.36	−11.0	<0.001	1.27 ± 0.12	−0.2	0.445
Manganese (mg)	3.6 ± 1.7	3.4 ± 1.7	3.8	0.074	4.5 ± 2.1	−20.3	<0.001	3.8 ± 1.7	4.6 ± 2.4	−16.3	<0.001	4.5 ± 0.7	0.3	0.165
Iodine (μg)	68.5 ± 55.9	64.1 ± 52.2	6.9	0.025	124.4 ± 53.7	−45.0	<0.001	70.2 ± 42.9	124.0 ± 49.1	−43.4	<0.001	124.4 ± 2.8	−0.1	0.578
Vitamin A (μg)	806 ± 447	780 ± 446	3.4	0.197	1065 ± 1 023	−24.3	<0.001	856 ± 413	1070 ± 999	−20.0	0.005	1067 ± 201	0.2	0.001
Retinol (μg)	401 ± 244	382 ± 258	5.0	0.007	465 ± 795	−13.7	0.838	402 ± 158	454 ± 789	−11.6	0.117	464 ± 59	−0.3	<0.001
β-carotene (μg)	2427 ± 2040	2384 ± 1974	1.8	0.397	3601 ± 3085	−32.6	<0.001	2722 ± 2333	3702 ± 3177	−26.5	<0.001	3611 ± 367	0.3	0.012
Vitamin D (μg)	3.5 ± 3.5	3.5 ± 3.7	0.2	0.026	3.5 ± 5.6	0.1	0.210	3.5 ± 2.7	3.4 ± 4.3	3.8	0.019	3.5 ± 0.6	−0.1	<0.001
Vitamin E (mg)	10.2 ± 6.0	9.7 ± 5.9	5.2	0.012	9.6 ± 5.0	5.5	0.827	10.1 ± 3.5	9.1 ± 3.1	11.0	0.002	9.6 ± 1.2	0.2	0.135
Vitamin B1 (mg)	1.21 ± 0.56	1.18 ± 0.56	3.0	0.151	1.33 ± 0.51	−8.7	0.004	1.25 ± 0.29	1.30 ± 0.37	−4.0	0.168	1.33 ± 0.15	−0.2	0.433
Vitamin B2 (mg)	1.64 ± 0.71	1.57 ± 0.73	5.0	0.002	1.87 ± 0.60	−12.2	<0.001	1.70 ± 0.40	1.89 ± 0.58	−9.9	<0.001	1.87 ± 0.16	−0.3	0.592
Niacin (mg)	20.6 ± 12.3	19.9 ± 11.3	3.4	0.502	22.1 ± 11.1	−6.8	0.017	20.6 ± 6.6	21.4 ± 8.0	−3.7	0.396	22.2 ± 2.9	0.2	0.186
Vitamin B6 (mg)	2.52 ± 2.08	2.34 ± 1.75	7.3	0.013	2.11 ± 0.86	18.9	0.486	2.41 ± 1.01	2.09 ± 0.66	15.5	0.003	2.11 ± 0.17	−0.2	0.198
Folates (μg)	227 ± 87	221 ± 93	2.6	0.039	301 ± 100	−24.7	<0.001	241 ± 66	303 ± 95	−20.6	<0.001	301 ± 24	0.1	0.205
Vitamin B12 (μg)	5.12 ± 3.70	4.79 ± 3.45	6.8	0.017	4.17 ± 3.01	22.7	<0.001	5.01 ± 2.07	4.14 ± 2.76	21.2	<0.001	4.16 ± 0.67	−0.2	0.002
Vitamin C (mg)	86.0 ± 48.2	83.9 ± 48.5	2.5	0.474	121.1 ± 68.6	−29.0	<0.001	93.2 ± 47.8	123.5 ± 74.9	−24.5	<0.001	121.0 ± 13.0	−0.1	0.287
Alcohol (g)	8.0 ± 9.8	8.1 ± 9.8	−2.2	0.308	4.4 ± 9.7	82.7	<0.001	7.8 ± 8.7	3.3 ± 7.0	138.3	<0.001	4.3 ± 2.4	−0.8	0.001
Protein (% energy)	17.5 ± 3.4	17.7 ± 3.4	−1.0	0.289	17.8 ± 3.9	−1.8	0.369	17.5 ± 3.4	17.8 ± 3.9	−1.8	0.374	17.6 ± 1.1	−1.6	0.659
Fat (% energy)	32.8 ± 5.7	32.3 ± 5.9	1.3	0.175	28.8 ± 6.5	13.7	<0.001	32.8 ± 5.7	28.8 ± 6.5	13.7	<0.001	29.6 ± 2.0	2.7	0.049
Carbohydrates (% energy)	48.7 ± 6.5	48.7 ± 6.7	−0.1	0.769	54.2 ± 7.0	−10.2	<0.001	48.7 ± 6.5	54.2 ± 7.0	−10.2	<0.001	53.7 ± 2.0	−0.9	0.218
Alcohol (% energy)	2.7 ± 3.1	2.9 ± 3.1	−6.6	0.018	1.1 ± 2.4	138.3	<0.001	2.7 ± 3.1	1.1 ± 2.4	138.3	<0.001	1.4 ± 0.7	23.9	<0.001

FFQcrude—crude data from the first administration of the FFQ (test), FFQretest—crude data from the second administration of the FFQ, FRcrude—crude data from the 2-day estimated food record (FR), FRstand—the FR data after standardization of energy intake, FFQstand—the FFQ data after standardization of energy intake, FFQreg—the FFQ data adjusted by regression, SD—standard deviation; *p*—the significance level of the Wilcoxon test, RD—relative difference (%) between mean values of energy and nutrient intake obtained with FFQ compared to the reference method (i.e., FFQcrude vs. FFQretest, FFQcrude vs. FRcrude, FFQstand vs. FRstand, FFQreg vs. FRcrude), SFA—saturated fatty acids, MUFA—monounsaturated fatty acids, PUFA—polyunsaturated fatty acids, LA—linoleic acid, ALA—α-linolenic acid, EPA—eicosapentaenoic acid, DHA—docosahexaenoic acid.

**Table 3:** Spearman’s correlation and intraclass correlation coefficient (FFQ reproducibility only) for energy and nutrient intake obtained with the 72-item SQ-FFQ and estimated food record (FR) among Polish young adults (*n* = 186).

Nutrient (Unit)	Reproducibility				Relative Validity					
	FFQcrude vs. FFQretest				FFQcrude vs. FRcrude		FFQstand vs. FRstand		FFQreg vs. FRcrude	
	r	*p*	ICC	*p*	r	*p*	r	*p*	r	*p*
Energy (kcal)	0.878	<0.001	0.917	<0.001	0.353	<0.001	-	-	0.353	<0.001
Total protein (g)	0.876	<0.001	0.867	<0.001	0.284	<0.001	0.247	0.001	0.286	<0.001
Animal protein (g)	0.856	<0.001	0.817	<0.001	0.246	0.001	0.218	0.003	0.246	0.001
Plant protein (g)	0.818	<0.001	0.856	<0.001	0.345	<0.001	0.294	<0.001	0.345	<0.001
Fat (g)	0.868	<0.001	0.930	<0.001	0.369	<0.001	0.376	<0.001	0.369	<0.001
SFA (g)	0.866	<0.001	0.907	<0.001	0.317	<0.001	0.294	<0.001	0.317	<0.001
MUFA (g)	0.865	<0.001	0.935	<0.001	0.390	<0.001	0.356	<0.001	0.391	<0.001
PUFA (g)	0.840	<0.001	0.906	<0.001	0.321	<0.001	0.108	0.143	0.321	<0.001
LA (g)	0.821	<0.001	0.883	<0.001	0.328	<0.001	0.130	0.077	0.325	<0.001
ALA (g)	0.867	<0.001	0.922	<0.001	0.314	<0.001	0.131	0.075	0.294	<0.001
EPA (mg)	0.776	<0.001	0.746	<0.001	0.030	0.680	0.049	0.503	0.031	0.675
DHA (mg)	0.799	<0.001	0.750	<0.001	0.127	0.085	0.136	0.065	0.127	0.084
Cholesterol (mg)	0.797	<0.001	0.825	<0.001	0.241	0.001	0.268	<0.001	0.242	0.001
Carbohydrates (g)	0.855	<0.001	0.874	<0.001	0.327	<0.001	0.312	<0.001	0.327	<0.001
Fructose (g)	0.703	<0.001	0.705	<0.001	0.299	<0.001	0.333	<0.001	0.299	<0.001
Sucrose (g)	0.852	<0.001	0.866	<0.001	0.295	<0.001	0.250	0.001	0.294	<0.001
Fiber (g)	0.811	<0.001	0.792	<0.001	0.281	<0.001	0.481	<0.001	0.281	<0.001
Sodium (mg)	0.855	<0.001	0.903	<0.001	0.337	<0.001	0.042	0.568	0.337	<0.001
Potassium (mg)	0.827	<0.001	0.830	<0.001	0.272	<0.001	0.287	<0.001	0.272	<0.001
Calcium (mg)	0.790	<0.001	0.827	<0.001	0.115	0.117	0.320	<0.001	0.115	0.117
Phosphorus (mg)	0.873	<0.001	0.846	<0.001	0.208	0.004	0.494	<0.001	0.208	0.004
Magnesium (mg)	0.826	<0.001	0.843	<0.001	0.246	0.001	0.533	<0.001	0.246	0.001
Iron (mg)	0.859	<0.001	0.890	<0.001	0.305	<0.001	0.274	<0.001	0.300	<0.001
Zinc (mg)	0.856	<0.001	0.873	<0.001	0.280	<0.001	0.376	<0.001	0.278	<0.001
Copper (mg)	0.833	<0.001	0.862	<0.001	0.294	<0.001	0.445	<0.001	0.265	<0.001
Manganese (mg)	0.794	<0.001	0.790	<0.001	0.301	<0.001	0.546	<0.001	0.301	<0.001
Iodine (μg)	0.808	<0.001	0.801	<0.001	−0.025	0.736	0.021	0.771	0.028	0.701
Vitamin A (μg)	0.693	<0.001	0.683	<0.001	0.069	0.350	0.265	<0.001	0.069	0.350
Retinol (μg)	0.799	<0.001	0.835	<0.001	0.177	0.016	0.184	0.012	0.177	0.016
β-carotene (μg)	0.631	<0.001	0.583	<0.001	0.202	0.006	0.350	<0.001	0.202	0.006
Vitamin D (μg)	0.820	<0.001	0.777	<0.001	0.209	0.004	0.171	0.020	0.218	0.003
Vitamin E (mg)	0.816	<0.001	0.886	<0.001	0.256	<0.001	0.168	0.022	0.255	<0.001
Vitamin B1 (mg)	0.836	<0.001	0.855	<0.001	0.283	<0.001	0.268	<0.001	0.271	<0.001
Vitamin B2 (mg)	0.848	<0.001	0.841	<0.001	0.290	<0.001	0.437	<0.001	0.297	<0.001
Niacin (mg)	0.837	<0.001	0.851	<0.001	0.330	<0.001	0.106	0.151	0.329	<0.001
Vitamin B6 (mg)	0.800	<0.001	0.777	<0.001	0.291	<0.001	0.058	0.430	0.279	<0.001
Folates (μg)	0.829	<0.001	0.838	<0.001	0.287	<0.001	0.427	<0.001	0.287	<0.001
Vitamin B12 (μg)	0.841	<0.001	0.819	<0.001	0.155	0.035	0.238	0.001	0.153	0.038
Vitamin C (mg)	0.721	<0.001	0.737	<0.001	0.248	0.001	0.197	0.007	0.248	0.001
Alcohol (g)	0.856	<0.001	0.854	<0.001	0.274	<0.001	0.254	<0.001	0.274	<0.001
Protein (% energy)	0.735	<0.001	0.759	<0.001	0.247	0.001	0.250	0.001	0.282	<0.001
Fat (% energy)	0.746	<0.001	0.744	<0.001	0.376	<0.001	0.375	<0.001	0.342	<0.001
Carbohydrates (% energy)	0.724	<0.001	0.690	<0.001	0.312	<0.001	0.314	<0.001	0.307	<0.001
Alcohol (% energy)	0.823	<0.001	0.666	<0.001	0.254	<0.001	0.257	<0.001	0.251	0.001

FFQcrude—crude data from the first administration of the FFQ (test), FFQretest—crude data from the second administration of the FFQ, FRcrude—crude data from the 2-day estimated food record (FR), FRstand—the FR data after standardization of energy intake, FFQstand—the FFQ data after standardization of energy intake, FFQreg—the FFQ data adjusted by regression, r—Spearman’s correlation coefficient, *p*—the significance level of the correlation, ICC—the intraclass correlation coefficient for test and retest of the FFQ, SFA—saturated fatty acids, MUFA—monounsaturated fatty acids, PUFA—polyunsaturated fatty acids, LA—linoleic acid, ALA—α-linolenic acid, EPA—eicosapentaenoic acid, DHA—docosahexaenoic acid.

**Table 4:** Cross-classification of quartiles of energy and nutrient intake obtained with the 72-item SQ-FFQ and estimated food record (FR) among Polish young adults (*n* = 186).

Nutrient (Unit)	Reproducibility			Relative Validity								
	FFQcrude vs. FFQretest			FFQcrude vs. FRcrude			FFQstand vs. FRstand			FFQreg vs. FRcrude		
	Same	Same and Adjacent	Extreme	Same	Same and Adjacent	Extreme	Same	Same and Adjacent	Extreme	Same	Same and Adjacent	Extreme
	%	%	%	%	%	%	%	%	%	%	%	%
Energy (kcal)	69.4	95.7	0.0	35.5	73.1	5.9	0.0	0.0	0.0	35.5	73.7	5.9
Total protein (g)	67.7	97.8	0.5	28.5	69.9	4.3	31.2	65.6	4.8	28.5	69.9	4.3
Animal protein (g)	65.1	96.8	0.0	24.7	67.2	5.4	29.0	69.4	7.0	24.7	67.2	5.4
Plant protein (g)	59.1	94.6	0.5	34.9	72.6	3.2	33.3	70.4	4.8	36.0	72.6	3.8
Fat (g)	67.2	95.7	0.0	32.8	71.5	5.4	36.6	77.4	5.9	32.3	71.5	5.4
SFA (g)	69.9	95.7	0.5	36.0	68.8	5.9	32.8	71.0	7.5	35.5	69.4	5.9
MUFA (g)	68.8	96.8	0.0	37.6	75.3	5.9	38.7	76.9	5.4	37.6	75.8	5.9
PUFA (g)	68.3	92.5	0.0	31.7	73.1	7.5	28.5	68.3	12.4	31.2	73.1	7.5
LA (g)	64.0	93.5	0.5	31.7	73.7	5.9	31.7	68.8	10.2	31.2	73.1	6.5
ALA (g)	65.6	95.7	0.0	37.1	73.7	5.4	24.7	68.3	10.2	37.1	74.2	5.4
EPA (mg)	69.9	89.8	0.5	23.7	61.3	11.8	24.7	63.4	11.3	23.1	61.3	10.8
DHA (mg)	67.2	93.0	0.5	31.7	66.7	10.8	33.9	65.6	6.5	31.7	66.7	10.2
Cholesterol (mg)	64.0	91.9	1.6	32.3	71.0	7.0	29.6	73.7	9.7	32.3	71.0	6.5
Carbohydrates (g)	66.7	96.8	0.0	32.3	72.6	5.9	37.6	74.7	7.0	31.7	72.6	5.9
Fructose (g)	58.1	87.6	1.1	34.9	73.7	5.9	36.0	77.4	8.6	34.4	73.7	6.5
Sucrose (g)	65.6	96.2	0.5	31.7	73.7	9.1	31.7	68.3	5.9	31.7	73.7	9.1
Fiber (g)	62.4	91.4	0.0	33.9	72.6	8.1	34.9	77.4	4.8	33.3	72.0	8.1
Sodium (mg)	67.2	97.3	0.0	31.2	71.0	5.9	28.0	68.3	14.0	31.2	71.0	5.9
Potassium (mg)	62.4	93.5	1.1	26.9	71.0	5.4	33.9	72.0	5.9	26.9	71.5	5.4
Calcium (mg)	61.8	90.3	0.0	30.1	64.0	10.2	36.0	71.0	4.3	29.6	63.4	10.2
Phosphorus (mg)	71.0	95.2	0.0	29.0	69.4	7.0	43.5	80.6	2.7	29.0	68.8	7.0
Magnesium (mg)	67.2	92.5	0.5	30.1	68.8	7.0	41.9	81.2	1.6	29.6	68.8	7.5
Iron (mg)	68.8	95.2	0.0	39.2	74.2	9.7	33.9	72.0	7.0	38.7	74.2	9.1
Zinc (mg)	66.1	96.2	0.5	31.7	74.7	8.6	34.4	74.7	6.5	31.2	74.7	8.1
Copper (mg)	64.5	94.6	0.0	30.6	72.6	5.9	38.2	75.8	3.2	29.0	74.2	6.5
Manganese (mg)	67.2	92.5	0.5	35.5	73.1	7.0	38.7	83.9	1.6	34.4	73.1	7.5
Iodine (μg)	60.8	95.7	0.0	23.1	62.9	11.8	27.4	64.0	12.9	22.6	60.2	10.2
Vitamin A (μg)	57.0	89.2	2.2	26.3	66.1	10.2	29.6	73.1	7.5	26.3	65.6	10.8
Retinol (μg)	60.8	92.5	0.5	30.6	69.4	9.7	27.4	69.4	8.6	31.2	69.9	9.7
β-carotene (μg)	58.1	84.9	2.7	30.1	71.5	7.5	30.1	74.7	5.4	30.1	71.5	7.5
Vitamin D (μg)	67.2	93.0	0.5	29.0	69.9	8.6	32.8	63.4	8.6	29.6	69.9	9.7
Vitamin E (mg)	66.1	92.5	0.5	26.3	68.3	5.9	30.1	68.3	9.7	26.9	68.8	5.9
Vitamin B1 (mg)	66.7	94.1	0.0	32.3	73.1	6.5	30.6	71.0	6.5	31.7	72.6	6.5
Vitamin B2 (mg)	64.5	96.8	0.0	36.0	70.4	6.5	33.3	75.3	4.3	34.9	69.9	6.5
Niacin (mg)	71.0	94.1	0.0	40.3	76.9	7.5	31.7	65.6	10.8	39.8	76.9	7.5
Vitamin B6 (mg)	66.1	93.5	1.1	33.3	72.6	6.5	30.1	65.1	14.0	33.3	73.1	6.5
Folates (μg)	62.9	96.8	1.1	32.8	75.3	8.1	37.6	75.8	4.8	32.8	75.3	8.1
Vitamin B12 (μg)	61.3	95.7	0.5	25.8	67.7	9.1	30.1	71.0	8.6	25.8	69.4	8.6
Vitamin C (mg)	63.4	89.2	1.1	28.0	71.0	4.3	29.0	68.8	8.6	28.0	71.0	4.3
Alcohol * (g)	74.2	95.7	0.0	-	-	-	-	-	-	-	-	-
Protein (% energy)	61.3	89.2	1.1	30.6	65.6	5.4	30.6	65.6	5.4	27.4	72.0	8.1
Fat (% energy)	57.5	88.7	0.5	36.6	77.4	5.9	36.6	77.4	5.9	33.9	78.5	6.5
Carbohydrates (% energy)	55.4	91.4	1.6	37.6	74.7	7.0	37.6	74.7	7.0	36.0	74.7	5.9
Alcohol * (% energy)	66.7	94.6	1.6	-	-	-	-	-	-	-	-	-

FFQcrude—crude data from the first administration of the FFQ (test), FFQretest—crude data from the second administration of the FFQ, FRcrude—crude data from the 2-day estimated food record (FR), FRstand—the FR data after standardization of energy intake, FFQstand—the FFQ data after standardization of energy intake, FFQreg—the FFQ data adjusted by regression, SFA—saturated fatty acids, MUFA—monounsaturated fatty acids, PUFA—polyunsaturated fatty acids, LA—linoleic acid, ALA—α-linolenic acid, EPA—eicosapentaenoic acid, DHA—docosahexaenoic acid, * cross-classification analysis between both methods was not performed due to the specific distribution of alcohol intake obtained with the FR.

## Data Availability

The data supporting the conclusions of this article will be made available from the corresponding author upon reasonable request.

## Supplementary Materials

The following supporting information can be downloaded at: https://www.mdpi.com/article/10.3390/nu14132696/s1, Table S1: The 72-item semi-quantitative food frequency questionnaire (72-item SQ-FFQ): Representatives of the food items and standard food portion sizes; Table S2: Regression equations for energy, nutrient and alcohol intake obtained with the 72-item SQ-FFQ.
