# Supplementary material for: The 72-Item Semi-Quantitative Food Frequency Questionnaire (72-Item SQ-FFQ) for Polish Young Adults: Reproducibility and Relative Validity
Source: Nutrients. 2022 Jun 29;14(13):2696. doi: 10.3390/nu14132696 (PMC9268558; doi:10.3390/nu14132696)
Supplement: Supplementary file 1 [file nutrients-14-02696-s001.zip › nutrients-1779775-supplementary.pdf]

nutrients

MDPI

Supplementary Materials

The 72-Item Semi-Quantitative Food Frequency Questionnaire (72-Item SQ-FFQ) for Polish Young Adults: Reproducibility and Relative Validity

Joanna Kowalkowska * and Lidia Wadolowska

Department of Human Nutrition, The Faculty of Food Science, University of Warmia and Mazury in Olsztyn, Słoneczna 45F, 10-718 Olsztyn, Poland; lidia.wadolowska@uwm.edu.pl
* Correspondence: joanna.kowalkowska@uwm.edu.pl; Tel.: +48-89-524-5517

Table S1. The 72-item semi-quantitative food frequency questionnaire (72-item SQ-FFQ): Representatives of the food items and standard food portion sizes

No.	Food item	Food examples	Representative of the food item	Standard portion size[1] [g]	Description of the portion size
1	Sugar	Sugar added to beverages, such as tea, coffee, etc.	Sugar	10	2 level teaspoons
2	Honey	Honey added to dishes and added to beverages	Honey	10	1 level teaspoon
3	Chocolates	Chocolate, chocolate sweets and chocolate bars	Milk chocolate	50	½ bar
4	Sugar confectionery	Boiled sweets, hard caramels, jelly sweets, fudge, etc.	Jelly sweets	25	½ handful (or ¼ of a small package)
5	Baked confectionery	Biscuits, cream cakes, fruit cakes, sponge cakes, cheesecakes, doughnuts, poppy-seed cakes, muffins, croissants, etc.	Biscuits	50	10 pieces (or 1 small package)
6	Ice creams and custard	Ice creams and custard	Ice cream	100	1 medium piece (or 2 scoops)
7	Savoury snacks	Crisps, crackers, pretzels, etc.	Potato crisps (with paprika)	70	½ medium package
8	Milk and milk beverages – natural	Milk and natural milk beverages (yoghurt, kefir, buttermilk), etc.	Milk (2.0% fat content)	200	1 glass (not a full)
9	Milk beverages – sweetened	Fruit yoghurts, yoghurts with chocolate flakes, flavoured buttermilk, hot chocolate, etc.	Yogurt with strawberries (1.5% fat content)	200	1 glass (not a full) or 1 medium package

No.	Food item	Food examples	Representative of the food item	Standard portion size[1] [g]	Description of the portion size
10	Cheese curds	Cheese curd, natural cottage cheese, soft cheese, mozzarella, cottage cheese with herbs, etc.	Quark, fresh cheese (semi-fat)	50	½ medium package
11	Flavoured cheese curds	Flavoured curds (with fruit, chocolate, vanilla), etc.	Fresh cheese with vanilla (homogenized)	150	1 small/medium package
12	Cheese	Hard cheese, blue cheese, processed cheese, cheese spreads, etc.	Gouda cheese	30	1 medium slice
13	Eggs and egg dishes	Scrambled eggs, omelette, egg salad, cooked eggs	Chicken eggs	100	2 medium pieces
14	Wholemeal bread	Wholemeal wheat or rye bread, seeded loafs, pumpernickel, wholemeal cracker bread, etc.	Dark rye bread	70	2 medium slices
15	White bread	White bread, rye, wheat-rye bread, toast bread, white bread rolls, brioche, bagels, etc.	Wheat-rye bread (white)	70	2 medium slices
16	Cereals	Oat flakes, barley flakes, rice flakes, muesli (not 'crunchy'), etc.	Oat flakes	40	4 tablespoons
17	Ready-to-eat breakfast cereals	Muesli crunchy, cornflakes, other ready-to-eat breakfast cereals (sweetened), etc.	Corn flakes	30	1 glass (10 tablespoons)
18	White pasta and white pasta dishes	Plain pasta, spaghetti, lasagne, etc.	White pasta (boiled)	200	2/3 dinner plate
19	Wholemeal pasta and wholemeal pasta dishes	Plain pasta, spaghetti, lasagne, etc.	Buckwheat pasta (boiled)	200	2/3 dinner plate
20	Coarse groats	Buckwheat groats, barley groats, brown rice, etc.	Buckwheat groats (boiled)	170	2/3 dinner plate
21	Fine groats	Semolina, milled barley, white rice, etc.	White rice (boiled)	170	2/3 dinner plate
22	Sweet dumplings and pancakes	Dumplings or pancakes with fruit, cottage cheese, etc.	Polish dumplings with fresh cheese (boiled)	320	8 medium pieces

No.	Food item	Food examples	Representative of the food item	Standard portion size[1] [g]	Description of the portion size
23	Savoury dumplings and pancakes	Dumplings or pancakes with meat, cabbage, mushrooms, etc.	Polish dumplings with meat (boiled)	320	8 medium pieces
24	Fast food	Pizza, hamburgers, kebab, etc.	Pizza with mushrooms and onion	300	½ medium pizza
25	Vegetable oil	All kinds of vegetable oils	Rapeseed oil	10	1 tablespoon
26	Butter	Butter	Butter	10	2 level teaspoons (1 single-serve package)
27	Margarine	Margarine for baking, frying, spreading	Margarine (70% fat content, with A and D)	10	2 level teaspoons
28	Cream	Single, double, sour, used as an ingredient or added to beverages	Cream (18% fat content)	20	1 tablespoon
29	Other animal fats	Lard, pork fat, etc.	Lard	10	2 level teaspoons
30	Mayonnaise and salad dressings	Mayonnaise, salad dressings based on mayonnaise, vinaigrette, etc.	Mayonnaise (from rapeseed oil)	20	1 tablespoon
31	All kinds of fruits	All kinds of fruits	Apple	150*	1 medium piece
32	Stone fruit	Apricots, cherries, nectarines, peaches, plums, grapes, etc.	Plums	150*	7 pieces
33	Kiwi and citrus fruit	Kiwi fruit, oranges, mandarins, grapefruit, lemons, pomelos, etc.	Kiwi fruit	100*	1 medium piece
34	Tropical fruits	Pineapples, watermelon, melons, fresh dates and figs, etc.	Pineapple	150	5 slices
35	Berries	Strawberries, raspberries, blackberries, blueberries, redcurrants, blackcurrants, etc.	Strawberries	150	1 glass
36	Bananas	Bananas	Banana	170*	1 medium piece
37	Apples and pears	Apples, pears	Apple	150*	1 medium piece
38	Avocado	Avocado	Avocado	140*	1 medium piece
39	Olives	Olives	Green olives (tinned)	25	10 pieces
40	Dried fruit	Raisins, dried apricots, figs, apples, prunes, etc.	Raisins	30	1 handful

No.	Food item	Food examples	Representative of the food item	Standard portion size[1] [g]	Description of the portion size
41	Fruit preserves and fruit condiments	Fruit compote, jams, marmalades, dates, dried bananas, etc.	Strawberries jam (high-sugar)	30	1 tablespoon
42	All kinds of vegetables (potatoes not included)	All kinds of vegetables (pota-toes not included)	White cabbage	100	1 small bowl
43	Cruciferous vegetables	Cabbages, Brussel sprouts, cauliflower, broccoli, kale, etc.	White cabbage	100	1 glass
44	Yellow-orange vegetables	Carrots, peppers, etc.	Carrot	100	2 medium pieces (1 glass of grated carrot)
45	Leafy green vegetables	Spinach, chicory, lettuce, rocket, leek, celery, parsley, etc.	Lettuce	50	2 handfuls
46	Tomatoes	Tomatoes	Tomato	130	1 medium piece
47	Gourds and squashes	Fresh cucumber, marrow, courgettes, pumpkins, aubergines, etc.	Cucumber	100	1 medium piece
48	Root vegetables and others	Parsnip, beetroots, onion, garlic, celeriac, radishes, turnip, salads and mixed vegetables, etc.	Beetroot	100	1 medium piece
49	Fresh and tinned legumes	Corn, green peas, green beans, etc.	Sweetcorn (tinned)	100	1 small plate
50	Dry and processed pulses	Beans, soya, peas, chickpeas, and processed pulses (baked beans, hummus, other bread spreads)	White beans (dried)	40	¼ glass
51	Potato chips, pancakes, rosti	Potato chips, potato pancakes, potato rosti, etc.	Potato chips (fried)	120	1 small plate
52	Potatoes	Boiled or baked potatoes	Potato (boiled)	300	4 medium pieces
53	Other potato dishes	Potato dumplings, gnocchi, etc.	Potato dumplings (boiled)	300	1 dinner plate
54	Nuts	Peanuts, hazelnuts, walnuts, cashews, coconuts, chestnuts, etc.	Peanuts	30	1 handful
55	Nut spreads	Peanut butter, chocolate-nut spread, etc.	Chocolate-nut spread	15	2 level teaspoons

No.	Food item	Food examples	Representative of the food item	Standard portion size[1] [g]	Description of the portion size
					(1 single-serve package)
56	Seeds and bran	Pumpkin seeds, sesame seeds, sunflower seeds, wheat germs, wheat bran, etc.	Sunflower seeds (dried)	30	1 handful
57	Hot-dogs and reconstituted meat	Hot-dogs, meat loaf, etc.	Wiener (pork)	100	2 medium pieces
58	Sausages	Smoked sausages, salami, etc.	Sausage (pork)	100	1 medium piece
59	High quality cured meats	Ham, poultry and pork-beef good quality cold meats, etc.	Ham (pork)	30	2 medium slice
60	Offal products	Liver, blood sausage, sweetbread, pate, bacon, etc.	Pâté (pork, roasted)	30	2 medium slices
61	Red meat	Pork, beef, veal, etc.	Pork neck (raw)	100	1 portion of the size of a deck of cards
62	Poultry and rabbit	Poultry, rabbit	Chicken breast (raw, without skin)	100	1 portion of the size of a deck of cards
63	Venison	Wild boar, roe deer, hare, etc.	Wild boar (raw)	100	1 portion of the size of a deck of cards
64	Lean fish	Pollock, cod, perch, hake, carp to 1 kg, tuna, panga, trout, etc.	Pollock (raw)	150	1 palm-sized portion
65	Oily fish	Salmon, sardines, herring, mackerel, eel, large carp, etc.	Salmon (raw)	150	1 palm-sized portion
66	Fruit juices and nectars	Mixed fruit juice, orange, grapefruit, apple, pear, grape, blackcurrant, cherry juice, etc.	Orange juice	200	1 glass (not a full)
67	Vegetable and vegetable-fruit juices	Mixed vegetable juice, tomato, carrot and carrot-fruit juice, etc.	Carrot, banana, and apple juice	200	1 glass (not a full)
68	Energy drinks	Energy drinks	Energy drinks	250	1 glass (or 1 can)
69	Sweetened beverages	Non-diet soft drinks/sodas, etc.	Sweetened beverages (cola)	250	1 glass
70	Beer	Beer	Beer	500	1 medium glass (or 1 can)

No.	Food item	Food examples	Representative of the food item	Standard portion size[1] [g]	Description of the portion size
71	Wine and cocktails	Wine and cocktails	Red wine	150	1 medium glass
72	Spirits	Vodka and other spirits	Spirits (40% volume)	50	1 medium/large glass<br>(or 2 shots)

[1] portion size universal for both men and women, *portion size of a market product (including waste); for other food products, portion sizes are given for the edible parts.

Table S2. Regression equations for energy, nutrient and alcohol intake obtained with the 72-item SQ-FFQ

Nutrient	a	b	Standard error	Regression equations[1]	R^2	*P*
Energy (kcal)	0.31	1472.58	687.61	y = 0.31 × x + 1 472.58 ± 687.61	0.154	<0.001
Total protein (g)	0.19	74.70	30.37	y = 0.19 × x + 74.70 ± 30.37	0.060	<0.001
Animal protein (g)	0.18	50.30	24.83	y = 0.18 × x + 50.30 ± 24.83	0.048	0.003
Plant protein (g)	0.26	22.13	9.30	y = 0.26 × x + 22.13 ± 9.30	0.106	<0.001
Fat (g)	0.33	44.58	29.56	y = 0.33 × x + 44.58 ± 29.56	0.176	<0.001
SFA (g)	0.34	17.04	11.64	y = 0.34 × x + 17.04 ± 11.64	0.150	<0.001
MUFA (g)	0.34	16.72	12.80	y = 0.34 × x + 16.72 ± 12.80	0.188	<0.001
PUFA (g)	0.23	7.64	5.37	y = 0.23 × x + 7.64 ± 5.37	0.095	<0.001
LA (g)	0.23	6.10	4.27	y = 0.23 × x + 6.10 ± 4.27	0.089	<0.001
ALA (g)	0.23	1.25	1.38	y = 0.23 × x + 1.25 ± 1.38	0.043	0.005
EPA (mg)	0.13	54.63	138.19	y = 0.13 × x + 54.63 ± 138.19	0.028	0.023
DHA (mg)	0.05	168.06	374.31	y = 0.05 × x + 168.06 ± 374.31	0.006	0.314
Cholesterol (mg)	0.27	262.62	201.54	y = 0.27 × x + 262.62 ± 201.54	0.056	0.001
Carbohydrates (g)	0.32	203.66	90.39	y = 0.32 × x + 203.66 ± 90.39	0.124	<0.001
Fructose (g)	0.60	8.96	7.85	y = 0.60 × x + 8.96 ± 7.85	0.067	<0.001
Sucrose (g)	0.31	34.86	30.17	y = 0.31 × x + 34.86 ± 30.17	0.084	<0.001
Fibre (g)	0.33	14.87	7.46	y = 0.33 × x + 14.87 ± 7.46	0.083	<0.001
Sodium (mg)	0.54	2378.22	1239.80	y = 0.54 × x + 2 378.22 ± 1 239.80	0.136	<0.001
Potassium (mg)	0.24	2776.73	1116.24	y = 0.24 × x + 2 776.73 ± 1 116.24	0.055	0.001
Calcium (mg)	0.15	707.24	328.88	y = 0.15 × x + 707.24 ± 328.88	0.023	0.041
Phosphorus (mg)	0.16	1245.95	464.72	y = 0.16 × x + 1 245.95 ± 464.72	0.040	0.006
Magnesium (mg)	0.24	272.77	116.86	y = 0.24 × x + 272.77 ± 116.86	0.066	<0.001
Iron (mg)	0.27	9.92	4.73	y = 0.27 × x + 9.92 ± 4.73	0.059	<0.001
Zinc (mg)	0.22	8.67	3.47	y = 0.22 × x + 8.67 ± 3.47	0.066	<0.001
Copper (mg)	0.25	1.00	0.43	y = 0.25 × x + 1.00 ± 0.43	0.080	<0.001
Manganese (mg)	0.42	2.98	1.98	y = 0.42 × x + 2.98 ± 1.98	0.112	<0.001
Iodine (µg)	-0.05	127.79	53.76	y = -0.05 × x + 127.79 ± 53.76	0.003	0.489
Vitamin A (µg)	0.45	704.53	1005.95	y = 0.45 × x + 704.53 ± 1 005.95	0.038	0.008
Retinol (µg)	0.24	367.21	795.37	y = 0.24 × x + 367.21 ± 795.37	0.006	0.311
β-carotene (µg)	0.18	3174.19	3072.40	y = 0.18 × x + 3 174.19 ± 3 072.40	0.014	0.114
Vitamin D (µg)	0.18	2.89	5.54	y = 0.18 × x + 2.89 ± 5.54	0.013	0.117
Vitamin E (mg)	0.20	7.61	4.86	y = 0.20 × x + 7.61 ± 4.86	0.057	0.001
Vitamin B_1 (mg)	0.26	1.01	0.49	y = 0.26 × x + 1.01 ± 0.49	0.082	<0.001
Vitamin B_2 (mg)	0.23	1.49	0.58	y = 0.23 × x + 1.49 ± 0.58	0.076	<0.001
Niacin (mg)	0.24	17.23	10.78	y = 0.24 × x + 17.23 ± 10.78	0.069	<0.001
Vitamin B_6 (mg)	0.08	1.91	0.85	y = 0.08 × x + 1.91 ± 0.85	0.038	0.008
Folates (µg)	0.28	237.95	97.77	y = 0.28 × x + 237.95 ± 97.77	0.058	<0.001
Vitamin B_{12} (µg)	0.18	3.24	2.95	y = 0.18 × x + 3.24 ± 2.95	0.049	0.002
Vitamin C (mg)	0.27	97.80	67.47	y = 0.27 × x + 97.80 ± 67.47	0.037	0.009
Alcohol (g)	0.25	2.33	9.39	y = 0.25 × x + 2.33 ± 9.39	0.066	<0.001

[1] formula: y = ax + b ± standard error; x – energy or nutrient intake obtained with the 72-item SQ-FFQ (FFQ_{crude}), y – energy or nutrient intake from the FFQ_{crude} adjusted by regression to data obtained with the 2-day estimated food record (FR_{crude}), R^2 – determination coefficient, *P* – the significance level, SFA – saturated fatty acids, MUFA – monounsaturated fatty acids, PUFA – polyunsaturated fatty acids, LA – linoleic acid, ALA – α-linolenic acid, EPA – eicosapentaenoic acid, DHA – docosahexaenoic acid.
